# Biallelic variants in *DNA2* cause microcephalic primordial dwarfism

**DOI:** 10.1002/humu.23776

**Published:** 2019-06-23

**Authors:** Žygimantė Tarnauskaitė, Louise S. Bicknell, Joseph A. Marsh, Jennie E. Murray, David A. Parry, Clare V. Logan, Michael B. Bober, Deepthi C. de Silva, Angela L. Duker, David Sillence, Carol Wise, Andrew P. Jackson, Olga Murina, Martin A. M. Reijns

**Affiliations:** ^1^ MRC Human Genetics Unit, MRC Institute of Genetics and Molecular Medicine University of Edinburgh Edinburgh United Kingdom; ^2^ Division of Genetics, Department of Pediatrics Nemours/Alfred I. duPont Hospital for Children Wilmington Delaware; ^3^ Department of Physiology, Faculty of Medicine University of Kelaniya Colombo Sri Lanka; ^4^ Discipline of Genomic Medicine, Faculty of Medicine and Health University of Sydney Sydney Australia; ^5^ Western Sydney Genetics Program Sydney Children's Hospitals Network Westmead Australia; ^6^ Sarah M. and Charles E. Seay Center for Musculoskeletal Research Texas Scottish, Rite Hospital for Children Dallas Texas; ^7^ McDermott Center for Human Growth and Development University of Texas, Southwestern Medical Center Dallas Texas; ^8^ Department of Orthopaedic Surgery University of Texas Southwestern Medical Center Dallas Texas; ^9^ Department of Pediatrics University of Texas Southwestern Medical Center Dallas Texas

**Keywords:** DNA repair, DNA replication, DNA2, growth, microcephalic primordial dwarfism

## Abstract

Microcephalic primordial dwarfism (MPD) is a group of rare single‐gene disorders characterized by the extreme reduction in brain and body size from early development onwards. Proteins encoded by MPD‐associated genes play important roles in fundamental cellular processes, notably genome replication and repair. Here we report the identification of four MPD individuals with biallelic variants in *DNA2*, which encodes an adenosine triphosphate (ATP)‐dependent helicase/nuclease involved in DNA replication and repair. We demonstrate that the two intronic variants (c.1764‐38_1764‐37ins(53) and c.74+4A>C) found in these individuals substantially impair *DNA2* transcript splicing. Additionally, we identify a missense variant (c.1963A>G), affecting a residue of the ATP‐dependent helicase domain that is highly conserved between humans and yeast, with the resulting substitution (p.Thr655Ala) predicted to directly impact ATP/ADP (adenosine diphosphate) binding by DNA2. Our findings support the pathogenicity of these variants as biallelic hypomorphic mutations, establishing *DNA2* as an MPD disease gene.

## 1 INTRODUCTION

Microcephalic primordial dwarfism (MPD) is an umbrella term for a group of rare monogenic disorders of extreme growth failure, characterised by marked microcephaly and short stature. MPD has been operationally defined in terms of both occipito–frontal circumference (OFC) and height being at least four standard deviations (*SD*) below the age‐ and sex‐matched population mean (Faivre et al., 2002; Klingseisen & Jackson, 2011), although less restrictive criteria to encompass individuals with milder growth restriction have also been used (Shaheen et al., 2018). MPD encompasses several phenotypically distinct Mendelian disorders, such as Seckel syndrome (Majewski, Goecke, & Opitz, 1982; Seckel, 1960), microcephalic osteodysplastic primordial dwarfism Type 1 and Type 2 (Majewski, Ranke, Schinzel, & Opitz, 1982; Majewski, Stoeckenius, Kemperdick, & Opitz, 1982), and Meier–Gorlin syndrome (Gorlin, Cervenka, Moller, Horrobin, & Witkop, 1975). A phenotype continuum between primary microcephaly and MPD is also established for a number of genes (Shaheen et al., 2018; Verloes, Drunat, Gressens, & Passemard, 1993).

Proteins encoded by MPD disease genes participate in essential cellular processes, including DNA replication (Bicknell, Bongers, et al., 2011; Bicknell, Walker, et al., 2011; Burrage et al., 2015; Fenwick et al., 2016; Guernsey et al., 2011; Logan et al., 2018; Vetro et al., 2017), DNA damage response signalling and DNA repair (Harley et al., 2016; Murray et al., 2014, 2015; O'Driscoll, Ruiz‐Perez, Woods, Jeggo, & Goodship, 2003; Ogi et al., 2012; Qvist et al., 2011; Reynolds et al., 2017). Collectively, variants in these genes are thought to cause disease by prolonging the cell cycle, with reduced cell proliferation resulting in a smaller number of cells throughout the body and brain, and therefore a smaller person (Klingseisen & Jackson, 2011).

The adenosine triphosphate (ATP)‐dependent helicase/nuclease DNA2 is a multifunctional enzyme, involved in various aspects of DNA replication and repair, including Okazaki fragment maturation during lagging strand synthesis (Ayyagari, Gomes, Gordenin, & Burgers, 2003; Bae, Bae, Kim, & Seo, 2001; Gloor, Balakrishnan, Campbell, & Bambara, 2012), DNA end resection during double‐strand break repair (Cejka et al., 2010; Karanja, Cox, Duxin, Stewart, & Campbell, 2012; Nimonkar et al., 2011; Niu et al., 2010; Sturzenegger et al., 2014), degradation of reversed replication forks to promote replication restart after genotoxic stress (Thangavel et al., 2015) and regulation of replication checkpoint activation (Duxin et al., 2012). Additionally, DNA2 has been implicated in mitochondrial DNA replication and repair (Duxin et al., 2009; Zheng et al., 2008). Most of the cellular functions of DNA2 have been attributed to its nuclease activity, whereas the role of DNA2 helicase activity long remained unclear. However, recently it was shown to act as an ATP‐dependent translocase to promote rapid DNA degradation during DNA end resection (Levikova, Pinto, & Cejka, 2017; Miller et al., 2017). Unsurprisingly, because of its importance for genome replication and stability, DNA2 is essential for mammalian embryonic development (Lin et al., 2013). A homozygous intronic *DNA2* variant was previously reported as the likely causal variant for two related individuals diagnosed clinically with Seckel syndrome. This variant was shown to cause aberrant splicing and reduced DNA2 protein levels in patient cells, with cellular phenotypes rescued by transient wild‐type DNA2 expression (Shaheen et al., 2014). Here, we report the identification of additional biallelic *DNA2* (NM_001080449.2; MIM# 601810) variants in four unrelated MPD patients. Using cellular splicing assays and molecular modeling we provide evidence that these result in partial loss of function of the essential DNA replication/repair protein it encodes. Our work, alongside the findings of Shaheen et al. (2014), therefore establishes a causal link between DNA2 deficiency and impaired growth.

## 2 RESULTS

**Figure 1:**
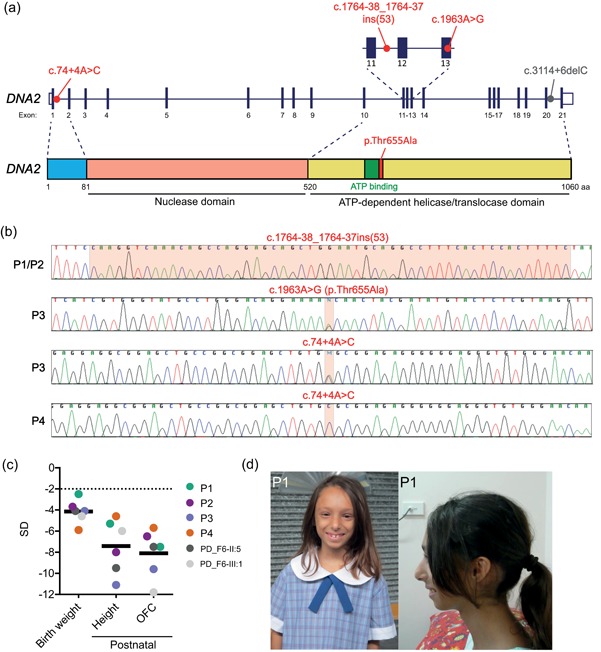
Identification of biallelic *DNA2* variants in microcephalic primordial dwarfism patients. (a) *DNA2* variants c.74+4A>C, c.1764‐38_1764‐37ins(53) and c.1963A>G (p.Thr655Ala, p.T655A) identified in four microcephalic primordial dwarfisms (MPD) patients are indicated in red on a schematic of the *DNA2* gene (NM_001080449.2), with the previously reported c.3114 + 6delC variant (Shaheen et al., 2014) shown in grey. Key domains and motifs are indicated on a cartoon model of the DNA2 protein (NP_001073918) structure. (b) Sanger sequencing chromatograms demonstrating *DNA2* variants (marked in red) in MPD patients. (c) Affected individuals exhibit extreme reduction in birth weight, postnatal height, and occipito–frontal circumference (OFC), reflecting global growth failure of prenatal onset and extreme microcephaly (this study: P1‐P4; Shaheen et al., 2014: PD_F6‐II:5, PD_F6‐III:1). Measurements plotted as *Z*‐scores (standard deviation, *SD*, of measurement from population mean for age and sex). Black bars represent the mean. (d) Photographs of P1. Written consent to publish photographs was obtained from the family

From WES sequencing of 192 MPD patients without a molecular diagnosis, we identified three unrelated patients (P1, P3, and P4) with biallelic variants in *DNA2* (Figure 1a and Table S1 and S2), with a fourth phenotypically similar patient (P2) identified through targeted resequencing of *DNA2* in our cohort. No likely causative variants in other genes were evident in WES datasets from P1, P3, and P4 (Tables S3 and S4). The two novel intronic variants (NM_001080449.2:c.1764‐38_1764‐37ins(53) and NM_001080449.2:c.74+4A>C) and single missense variant (NM_001080449.2:c.1963A>G, p.Thr655Ala) were validated using Sanger sequencing (Figure 1b). Parents of the affected individuals were heterozygous for these variants, and segregation in unaffected siblings from P1 and P3 consistent with an autosomal recessive inherited disorder. None of the variants were present in the gnomAD (genome aggregation) database (Lek et al., 2016), establishing these alleles to be very infrequent in the general population, in keeping with a rare Mendelian disorder. All variants were submitted to the LOVD Global Variome database (https://databases.lovd.nl/shared/genes/DNA2).

The patients exhibited severe microcephaly with OFC ranging from −5.7 *SD* to −9.6 *SD*, as well as markedly reduced height, ranging from −4.6 *SD* to −11.1 *SD* (Table S1, Figure 1c). Three out of four patients with *DNA2* variants were noted to have prominent, large upper incisors and a high frontal hairline, however a common facial gestalt was not otherwise apparent (Table S2, Figure 1d). No significant health problems were noted, aside from P3 who had severe thoracic kyphoscoliosis and recurrent chest infections (Table S2). All had normal intellectual development, with P3 and P4 now adolescents and P2 healthy when last met aged 46. Normal cognition, lack of a sloping forehead and proportionate OFC to height reduction led us to clinically classify these patients as MPD, rather than Seckel syndrome. Given the overlapping clinical phenotypes, we concluded that these variants were likely to be pathogenic, despite their predominantly intronic nature, and we next investigated the consequences of these variants on *DNA2* messenger RNA (mRNA) splicing.

**Figure 2:**
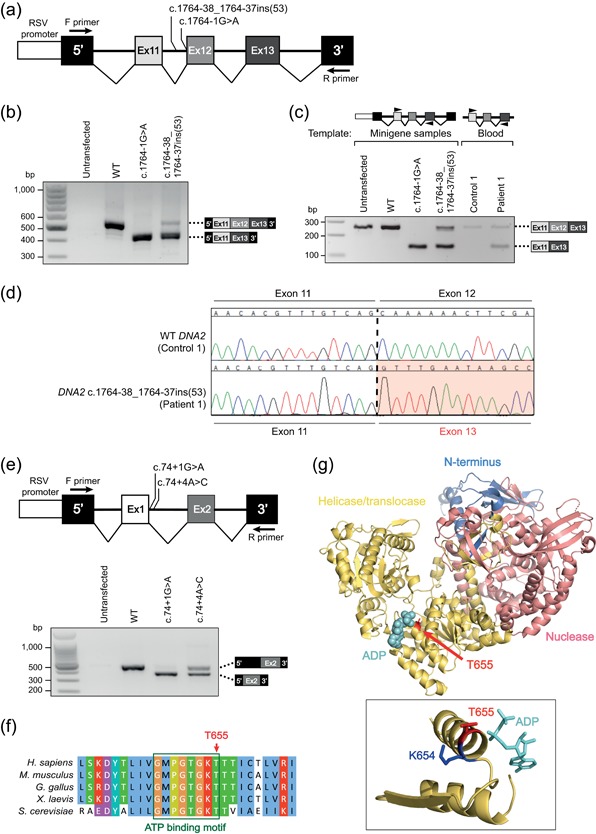
Transcriptional and structural consequences of *DNA2* variants identified in MPD patients. (a) RHCglo minigene reporter constructs used in the minigene splicing assay to assess the effect of the *DNA2* c.1764‐38_1764‐37ins(53) variant. A positive control for splicing disruption was generated by introducing a point mutation, abolishing the acceptor splice site of *DNA2* intron 11. Arrows indicate primers used for RT‐PCR analysis. (b) c.1764‐38_1764‐37ins(53) affects splicing of *DNA2* transcript. HeLa cells were transfected with minigene constructs, followed by RNA extraction, cDNA generation, and RT‐PCR analysis to assess *DNA2* splicing patterns. RT‐PCR products amplified from the HeLa cDNA of the minigene assay samples using F and R primers (see panel a) were separated by agarose gel electrophoresis. Sanger sequencing analysis of cloned PCR products revealed that the product with higher electrophoretic mobility represents transcript lacking exon 12, and the lower mobility product the correctly spliced transcript. (c) The splicing defect caused by *DNA2* c.1764‐38_1764‐37ins(53) is also detected in patient (P1) PBLs. PCR was performed using primers located in exon 11 and exon 13 of *DNA2* (indicated by short black arrows) products using cDNA generated from minigene assay samples and from RNA extracted from P1 peripheral blood. Agarose gel electrophoresis and Sanger sequencing analysis of PCR products recapitulated the previous findings. (d) Sanger electropherograms demonstrate that the c.1764‐38_1764‐37ins(53) variant results in skipping of *DNA2* exon 12. (e) Top: RHCglo minigene reporter constructs used in the minigene splicing assay to assess the effect of the *DNA2* c.74+4A>C variant. As a control, the c.74+1G>A point mutation was introduced, abolishing the acceptor splice site of *DNA2* intron 1. Bottom: *DNA2* c.74+4A>C variant affects splicing of *DNA2* transcript. PCR products amplified from HeLa cDNA of the minigene assay samples were separated by agarose gel electrophoresis. Sanger sequencing analysis of cloned PCR products revealed that the product with higher electrophoretic mobility represents transcript lacking exon 1 and the lower mobility product the correctly spliced transcript. (f) DNA2 threonine 655, mutated in MPD patient P3, is a highly conserved residue in the ATP binding motif (boxed in green) within the helicase/translocase domain. Alignment generated and visualized using Jalview multiple sequence alignment software (Waterhouse, Procter, Martin, Clamp, & Barton, 2009). (g) DNA2 p.K654 and p.T655 are important for ATP/ADP binding. Top: DNA2 protein domains (N‐terminus, nuclease and helicase/translocase) are represented in different colors (PDB ID: 5EAW; [Zhou et al., 2015]). ADP is shown in cyan spheres; p.T655, shown in red, indicates mouse DNA2 p.T656 (the equivalent of human p.T655). Below: this threonine residue contacts ADP, similar to p.K654 (p.K655 in mouse, indicated in blue = p.K654 in human DNA2). Substitution of K654 abolishes DNA2 ATPase activity. ADP, adenosine diphosphate; ATP, adenosine triphosphate; cDNA, complementary DNA; MPD, microcephalic primordial dwarfism; PBL, peripheral blood leukocytes; RT‐PCR, reverse‐transcription polymerase chain reaction

P1 and P2, not knowingly related, were homozygous for the same variant, a 53 bp insertion in the middle of the small (78 bp) intron 11 of *DNA2* (c.1764‐38_1764‐37ins(53); Figure 1a and Table S1). SpliceSiteFinder‐like, MaxEntScan, and Human Splicing Finder algorithms (Alamut Visual) predicted the creation of a new splice donor site after the first four bases of inserted sequence, suggesting that *DNA2* transcript splicing could be affected by this variant. As patient‐derived cell lines were not available and attempts to generate a lymphoblastoid cell line from P1 peripheral blood leukocytes (PBLs) were not successful, we used a minigene‐based splicing assay to study the consequences and establish the pathogenicity of this variant. Minigene splicing reporters (Singh & Cooper, 2006) containing the genomic region covering *DNA2* exon 11 to exon 13 from a healthy control and P1 were constructed (Figure 2a). While the splicing control with a disrupted acceptor splice site of intron 11 (c.1764‐1 G>A) demonstrated complete abrogation of correct splicing, the c.1764‐38_1764‐37ins(53) variant resulted in marked, but partial loss of correct splicing (Figure 2b). *DNA2* transcript analysis using RNA isolated from P1 PBLs also demonstrated altered splicing (Figure 2c), confirming that partial loss of correct *DNA2* splicing occurs as a result of this intronic variant. Capillary sequencing of reverse‐transcription polymerase chain reaction (RT‐PCR) products demonstrated correctly spliced mRNA for the wild‐type control, with *DNA2* exons 11, 12, and 13 included (Figure 2b–d), while for P1, the majority contained full‐length exons 11 and 13, but lacked exon 12 (Figure 2b–d), in keeping with the c.1764‐38_1764‐37ins(53) insertion causing substantial skipping of exon 12. Absence of exon 12 causes a frameshift leading to a premature termination codon in exon 13 (p.Ser588ArgfsTer4), which would promote degradation of such transcripts by nonsense‐mediated decay and/or lead to the translation of a severely truncated protein missing the majority of the helicase domain, including the ATP binding site. Both would result in marked reduction of cellular levels of functional DNA2 protein.

P4 was homozygous for a putative splice site variant in intron 1 (c.74 + 4A>C; Figure 1a and Table S1), predicted to be potentially deleterious (CADD score = 16.4). Furthermore, splice prediction analysis suggested that this variant would weaken the donor splice site of intron 1, and therefore result in aberrant splicing of *DNA2*. This was confirmed experimentally using another minigene assay (Figure 2e). Here, the c.74+4A>C mutant exhibited two RT‐PCR products that corresponded to correctly spliced and incorrectly spliced transcript lacking canonical exon 1, respectively. Nevertheless, as a result of residual levels of correct splicing for both the c.74+4A>C and c.1764‐38_1764‐37ins(53) variants, low levels of wild‐type transcript and protein would still be produced, in keeping with DNA2 function being essential for mammalian development (Lin et al., 2013).

Lastly, patient P3, who has the most severe reduction in height and the smallest head circumference (Table S1), was found to be compound heterozygous for the same c.74+4A>C variant *in trans* with a missense variant, c.1963A>G (p.Thr655Ala, p.T655A). P2 and P3 were phenotypically similar and not knowingly related, thus providing additional evidence to support pathogenicity of the c.74+4A>C variant. Likewise, there was strong evidence for the pathogenicity of the p.T655A substitution: the CADD score was 28.2, and given that threonine residue 655 is conserved to yeast and forms part of the ATP‐binding motif of the DNA2 helicase/translocase domain (Figures 1a, 2f), it was likely to be functionally critical. Furthermore, substitution of the neighboring lysine 654 residue (corresponding to lysine 671 in older literature based on previous nomenclature) abolishes DNA2 ATPase activity, reducing the speed of ssDNA degradation (Levikova et al., 2017; Masuda‐Sasa, Imamura, & Campbell, 2006). Therefore, substitution of the adjacent p.T655 might also be expected to affect hydrolysis of ATP, reducing the rate of translocation of DNA2 along DNA, consequently diminishing nuclease‐dependent degradation of ssDNA, and negatively impacting on DNA end resection. To investigate this possibility further, the crystal structure of mouse DNA2 (80% overall sequence identity to human DNA2; 100% in the ATP‐binding motif) bound to the ATP hydrolysis product, adenosine diphosphate (ADP; Zhou, Pourmal, & Pavletich, 2015) was examined.

Molecular modeling of the p.T656A variant (the mouse equivalent of human p.T655A) with FoldX (Guerois, Nielsen, & Serrano, 2002; Schymkowitz et al., 2005) predicted a negligible effect of the variant on intramolecular protein stability (ΔΔ*G* = −0.15 kcal/mol). However, the affected threonine residue forms substantial intermolecular contacts with ADP, burying 25.1 Å^2^ of solvent‐accessible surface area, supporting modulation of this interaction as the basis for pathogenicity of this substitution. Notably, analysis with mCSM‐lig (Pires, Blundell, & Ascher, 2016) predicted that the p.T656A variant weakens the interaction with ADP (by ~20%). This change in affinity would be expected to reduce DNA2 helicase activity and processivity.

However, as the DNA2 structure does not contain the magnesium divalent cation required for ATPase activity, we next examined homologous structures that contained both Mg^2+^ and ADP. For this we used crystal structures of human UPF1 (Chakrabarti et al., 2011) and *Saccharomyces cerevisiae* SEN1 helicases (Leonaite et al., 2017), both of which are highly homologous to DNA2 around the ATP‐binding site, at the amino acid and structural level (Figure S1A,B). Here, a substantially stronger reduction in ADP binding affinity as a result of the threonine to alanine change was predicted (2.6‐ to 3.1‐fold), likely due to contacts between the threonine residue and Mg^2+^ at the ATPase site (Figure S1B,C). Furthermore, molecular modelling of substitutions at the adjacent lysine residue predicted these to similarly weaken the interaction with ADP (Figure S1C). Notably, substitution of this lysine residue in DNA2 has previously been shown to abrogate ATPase activity (Levikova et al., 2017; Masuda‐Sasa et al., 2006). Therefore, while structural modelling of mutational effects on protein–ligand interactions has limitations, this molecular modelling, in conjunction with the direct physical contact of this threonine residue with ADP/Mg^2+^, suggests that p.T655A negatively affects cellular DNA2 enzyme function in the same manner as p.K654E/R, reducing processivity during DNA end resection.

## 3 DISCUSSION

Our identification of four unrelated patients with biallelic *DNA2* variants provides strong genetic evidence for *DNA2* as an MPD disease gene. As such, this substantively confirms the conclusions of Shaheen et al., who reported the homozygous c.3114+6delC intronic *DNA2* variant, found within a region of homozygosity, as the likely cause of Seckel syndrome in an extended consanguineous Saudi Arabian family (Shaheen et al., 2014). The term “Seckel syndrome” has frequently been used to describe patients with a sloping forehead, prominent nose, and intellectual disability, alongside disproportionate microcephaly (Hall, Flora, Scott, Pauli, & Tanaka, 2004; Kalay et al., 2011; Majewski, Goecke, et al., 1982). Individuals from the originally reported family were reported as having “Seckel‐like” facies (Shaheen et al., 2014); however, our cases do not have such an appearance. Consequently, we suggest employing the broader term “MPD” to categorize the phenotype of individuals with biallelic *DNA2* variants.

Heterozygous missense variants in *DNA2* have been associated with mitochondrial myopathy, as 'adult‐onset autosomal dominant progressive external ophthalmoplegia and mitochondrial DNA deletions type 6' (PEOA6; Ronchi et al., 2013). The level of DNA2 deficiency could account for the very different phenotypes Marked impairment in cellular DNA2 activity in MPD, resulting from biallelic *DNA2* variants, would be consistent with the necessity of adequate DNA2 protein levels during embryonic development, particularly during rapid cell proliferation, with a disruption in timely nuclear DNA replication/repair leading to fewer cells being generated, resulting in a smaller individual. In contrast, haploinsufficiency would be developmentally tolerated, but in the long term could result in mitochondrial DNA depletion and adult‐onset myopathy.

However, a simple model of differing levels of deficiency is difficult to reconcile with the lack of any features associated with mitochondrial disease in any of the six individuals with biallelic variants in *DNA2* (our study and Shaheen et al., 2014) or their carrier parents. This argues against a simple DNA2 “dosage‐effect” model to account for distinct mitochondrial and growth phenotypes. Furthermore, heterozygous *DNA2* truncating variants appear to be tolerated in the general population (gnomAD; Lek et al., 2016). Such population data seemingly runs counter to two case reports associating truncating variants with childhood myopathy (Chae et al., 2015; Phowthongkum & Sun, 2017), however, both studies failed to assess parental variant status or demonstrate mitochondrial DNA deletions, rendering their findings inconclusive. Therefore, as proposed by Shaheen et al., the PEOA6 *DNA2* variants (Ronchi et al., 2013) may instead have an allele‐specific effect. The biochemical findings of Ronchi et al were most consistent with decreased nuclease activity, whereas the p.T655A variant we identified in the helicase domain is expected to specifically impair end resection activity of DNA2 (Daley et al., 2017). This raises the possibility that differing functional consequences on this multifunctional enzyme could account for the different phenotypic outcomes in MPD and PEOA6. Notably, variants in *RBBP8*, which encodes CtIP, another DNA end resection factor, also lead to MPD (Qvist et al., 2011).

In conclusion, our findings in conjunction with the work of Shaheen et al., establish *DNA2* as an MPD gene. Future studies will be important to establish the molecular and cellular basis for the differing phenotypes of PEOA6 DNA2 and MPD‐DNA2, with ascertainment of further cases, derivation of patient cell lines and development of relevant animal models, important to distinguish between potential disease mechanisms.

## CONFLICT OF INTERESTS

The authors declare that there is no conflict of interests.

## Supporting information

Supporting information

Supporting information

## References

[humu23776-bib-0001] Ayyagari, R. , Gomes, X. V. , Gordenin, D. A. , & Burgers, P. M. J. (2003). Okazaki fragment maturation in yeast: I. Distribution of functions between FEN1 and DNA2. Journal of Biological Chemistry, 278(3), 1618–1625. 10.1074/jbc.M209801200 12424238

[humu23776-bib-0002] Bae, S. H. , Bae, K. H. , Kim, J. A. , & Seo, Y. S. (2001). RPA governs endonuclease switching during processing of Okazaki fragments in eukaryotes. Nature, 412(6845), 456–461. 10.1038/35086609 11473323

[humu23776-bib-0003] Bicknell, L. S. , Bongers, E. M. H. F. , Leitch, A. , Brown, S. , Schoots, J. , Harley, M. E. , … Jackson, A. P. (2011). Mutations in the pre‐replication complex cause Meier–Gorlin syndrome. Nature Genetics, 43(4), 356–360. 10.1038/ng.775 21358632 PMC3068194

[humu23776-bib-0004] Bicknell, L. S. , Walker, S. , Klingseisen, A. , Stiff, T. , Leitch, A. , Kerzendorfer, C. , … Jeggo, P. A. (2011). Mutations in ORC1, encoding the largest subunit of the origin recognition complex, cause microcephalic primordial dwarfism resembling Meier–Gorlin syndrome. Nature Genetics, 43(4), 350–356. 10.1038/ng.776 21358633

[humu23776-bib-0005] Burrage, L. C. , Charng, W. L. , Eldomery, M. K. , Willer, J. R. , Davis, E. E. , Lugtenberg, D. , … Yang, Y. (2015). De novo GMNN mutations cause autosomal‐dominant primordial dwarfism associated with Meier‐Gorlin syndrome. American Journal of Human Genetics, 97(6), 904–913. 10.1016/j.ajhg.2015.11.006 26637980 PMC4678788

[humu23776-bib-0006] Cejka, P. , Cannavo, E. , Polaczek, P. , Masuda‐Sasa, T. , Pokharel, S. , Campbell, J. L. , & Kowalczykowski, S. C. (2010). DNA end resection by Dna2‐Sgs1‐RPA and its stimulation by Top3‐Rmi1 and Mre11‐Rad50‐Xrs2. Nature, 467(7311), 112–116. 10.1038/nature09355.DNA 20811461 PMC3089589

[humu23776-bib-0007] Chae, J. H. , Vasta, V. , Cho, A. , Lim, B. C. , Zhang, Q. , Eun, S. H. , & Hahn, S. H. (2015). Utility of next generation sequencing in genetic diagnosis of early onset neuromuscular disorders. Journal of Medical Genetics, 52(3), 208–216. 10.1136/jmedgenet-2014-102819 25635128

[humu23776-bib-0008] Chakrabarti, S. , Jayachandran, U. , Bonneau, F. , Fiorini, F. , Basquin, C. , Domcke, S. , … Conti, E. (2011). Molecular mechanisms for the RNA‐dependent ATPase activity of Upf1 and its regulation by Upf2. Molecular Cell, 41(6), 693–703. 10.1016/j.molcel.2011.02.010 21419344

[humu23776-bib-0009] Daley, J. M. , Jimenez‐Sainz, J. , Wang, W. , Miller, A. S. , Xue, X. , Nguyen, K. A. , … Sung, P. (2017). Enhancement of BLM‐DNA2‐mediated long‐range DNA end resection by CtIP. Cell Reports, 21(2), 324–332. 10.1016/j.celrep.2017.09.048 29020620 PMC5689478

[humu23776-bib-0010] Duxin, J. P. , Dao, B. , Martinsson, P. , Rajala, N. , Guittat, L. , Campbell, J. L. , … Stewart, S. A. (2009). Human Dna2 is a nuclear and mitochondrial DNA maintenance protein. Molecular and Cellular Biology, 29(15), 4274–4282. 10.1128/MCB.01834-08 19487465 PMC2715806

[humu23776-bib-0011] Duxin, J. P. , Moore, H. R. , Sidorova, J. , Karanja, K. , Honaker, Y. , Dao, B. , … Stewart, S. A. (2012). Okazaki fragment processing‐independent role for human Dna2 enzyme during DNA replication. Journal of Biological Chemistry, 287(26), 21980–21991. 10.1074/jbc.M112.359018 22570476 PMC3381158

[humu23776-bib-0012] Faivre, L. , Le Merrer, M. , Lyonnet, S. , Plauchu, H. , Dagoneau, N. , Campos‐Xavier, A. B. , … Cormier‐Daire, V. (2002). Clinical and genetic heterogeneity of Seckel syndrome. American Journal of Medical Genetics, 112(4), 379–383. 10.1002/ajmg.10677 12376940

[humu23776-bib-0013] Fenwick, A. L. , Kliszczak, M. , Cooper, F. , Murray, J. , Sanchez‐Pulido, L. , Twigg, S. R. F. , … Bicknell, L. S. (2016). Mutations in CDC45, encoding an essential component of the pre‐initiation complex, cause Meier‐Gorlin syndrome and craniosynostosis. American Journal of Human Genetics, 99(1), 125–138. 10.1016/j.ajhg.2016.05.019 27374770 PMC5005452

[humu23776-bib-0014] Gloor, J. W. , Balakrishnan, L. , Campbell, J. L. , & Bambara, R. A. (2012). Biochemical analyses indicate that binding and cleavage specificities define the ordered processing of human Okazaki fragments by DNA2 and FEN1. Nucleic Acids Research, 40(14), 6774–6786. 10.1093/nar/gks388 22570407 PMC3413157

[humu23776-bib-0015] Gorlin, R. J. , Cervenka, J. , Moller, K. , Horrobin, M. , & Witkop, C. J. (1975). Malformation syndromes. A selected miscellany. Birth Defects Original Article Series, 11(2), 39–50. 819054

[humu23776-bib-0016] Guernsey, D. L. , Matsuoka, M. , Jiang, H. , Evans, S. , MacGillivray, C. , Nightingale, M. , … Samuels, M. E. (2011). Mutations in origin recognition complex gene ORC4 cause Meier–Gorlin syndrome. Nature Genetics, 43(4), 360–365. 10.1038/ng.777 21358631

[humu23776-bib-0017] Guerois, R. , Nielsen, J. E. , & Serrano, L. (2002). Predicting changes in the stability of proteins and protein complexes: A study of more than 1000 mutations. Journal of Molecular Biology, 320(2), 369–387. 10.1016/S0022-2836(02)00442-4 12079393

[humu23776-bib-0018] Hall, J. G. , Flora, C. , Scott, C. I. , Pauli, R. M. , & Tanaka, K. I. (2004). Majewski osteodysplastic primordial dwarfism type II (MOPD II): Natural history and clinical findings. American Journal of Medical Genetics, 130A(1), 55–72. 10.1002/ajmg.a.30203 15368497

[humu23776-bib-0019] Harley, M. E. , Murina, O. , Leitch, A. , Higgs, M. R. , Bicknell, L. S. , Yigit, G. , … Jackson, A. P. (2016). TRAIP promotes DNA damage response during genome replication and is mutated in primordial dwarfism. Nature Genetics, 48(1), 36–43. 10.1038/ng.3451 26595769 PMC4697364

[humu23776-bib-0020] Kalay, E. , Yigit, G. , Aslan, Y. , Brown, K. E. , Pohl, E. , Bicknell, L. S. , … Wollnik, B. (2011). CEP152 is a genome maintenance protein disrupted in Seckel syndrome. Nature Genetics, 43(1), 23–26. 10.1038/ng.725 21131973 PMC3430850

[humu23776-bib-0021] Karanja, K. K. , Cox, S. W. , Duxin, J. P. , Stewart, S. A. , & Campbell, J. L. (2012). DNA2 and EXO1 in replication‐coupled, homology‐directed repair and in the interplay between HDR and the FA/BRCA network. Cell Cycle, 11(21), 3983–3996. 10.4161/cc.22215 22987153 PMC3507494

[humu23776-bib-0022] Klingseisen, A. , & Jackson, A. P. (2011). Mechanisms and pathways of growth failure in primordial dwarfism. Genes and Development, 25(19), 2011–2024. 10.1101/gad.169037 21979914 PMC3197200

[humu23776-bib-0023] Lek, M. , Karczewski, K. J. , Minikel, E. V. , Samocha, K. E. , Banks, E. , Fennell, T. , … Exome Aggregation, C. (2016). Analysis of protein‐coding genetic variation in 60,706 humans. Nature, 536(7616), 285–291. 10.1038/nature19057 27535533 PMC5018207

[humu23776-bib-0024] Leonaite, B. , Han, Z. , Basquin, J. , Bonneau, F. , Libri, D. , Porrua, O. , & Conti, E. (2017). Sen1 has unique structural features grafted on the architecture of the Upf1‐like helicase family. EMBO Journal, 36(11), 1590–1604. 10.15252/embj.201696174 28408439 PMC5452015

[humu23776-bib-0025] Levikova, M. , Pinto, C. , & Cejka, P. (2017). The motor activity of DNA2 functions as an ssDNA translocase to promote DNA end resection. Genes and Development, 31(5), 493–502. 10.1101/gad.295196.116 28336515 PMC5393063

[humu23776-bib-0026] Lin, W. , Sampathi, S. , Dai, H. , Liu, C. , Zhou, M. , Hu, J. , … Shen, B. (2013). Mammalian Dna2 helicase/nuclease cleaves G‐quadruplex DNA and is required for telomere integrity. EMBO Journal, 32(10), 1425–1439. 10.1038/emboj.2013.88 23604072 PMC3655473

[humu23776-bib-0027] Logan, C. V. , Murray, J. E. , Parry, D. A. , Robertson, A. , Bellelli, R. , Tarnauskaitė, Ž. , … Jackson, A. P. (2018). DNA polymerase epsilon deficiency causes IMAGe syndrome with variable immunodeficiency. The American Journal of Human Genetics, 103, 1038–1044. 10.1016/j.ajhg.2018.10.024 30503519 PMC6288413

[humu23776-bib-0028] Majewski, F. , Goecke, T. , & Opitz, J. M. (1982). Studies of microcephalic primordial dwarfism I: Approach to a delineation of the Seckel syndrome. American Journal of Medical Genetics, 12(1), 7–21. 10.1002/ajmg.1320120103 7046443

[humu23776-bib-0029] Majewski, F. , Ranke, M. , Schinzel, A. , & Opitz, J. M. (1982). Studies of microcephalic primordial dwarfism II: The osteodysplastic type II of primordial dwarfism. American Journal of Medical Genetics, 12(1), 23–35. 10.1002/ajmg.1320120104 7201238

[humu23776-bib-0030] Majewski, F. , Stoeckenius, M. , Kemperdick, H. , & Opitz, J. M. (1982). Studies of microcephalic primordial dwarfism III: An intrauterine dwarf with platyspondyly and anomalies of pelvis and clavicles—Osteodysplastic primordial dwarfism type III. American Journal of Medical Genetics, 12(1), 37–42. 10.1002/ajmg.1320120105 7201239

[humu23776-bib-0031] Masuda‐Sasa, T. , Imamura, O. , & Campbell, J. L. (2006). Biochemical analysis of human Dna2. Nucleic Acids Research, 34(6), 1865–1875. 10.1093/nar/gkl070 16595800 PMC1428797

[humu23776-bib-0032] Miller, A. S. , Daley, J. M. , Pham, N. T. , Niu, H. , Xue, X. , Ira, G. , & Sung, P. (2017). A novel role of the Dna2 translocase function in DNA break resection. Genes and Development, 31(5), 503–510. 10.1101/gad.295659.116 28336516 PMC5393064

[humu23776-bib-0033] Murray, J. E. , Bicknell, L. S. , Yigit, G. , Duker, A. L. , van Kogelenberg, M. , Haghayegh, S. , … Jackson, A. P. (2014). Extreme growth failure is a common presentation of ligase IV deficiency. Human Mutation, 35(1), 76–85. 10.1002/humu.22461 24123394 PMC3995017

[humu23776-bib-0034] Murray, J. E. , Van Der Burg, M. , Ijspeert, H. , Carroll, P. , Wu, Q. , Ochi, T. , … Bicknell, L. S. (2015). Mutations in the NHEJ component XRCC4 cause primordial dwarfism. American Journal of Human Genetics, 96(3), 412–424. 10.1016/j.ajhg.2015.01.013 25728776 PMC4375537

[humu23776-bib-0035] Nimonkar, A. V. , Genschel, J. , Kinoshita, E. , Polaczek, P. , Campbell, J. L. , Wyman, C. , … Kowalczykowski, S. C. (2011). BLM‐DNA2‐RPA‐MRN and EXO1‐BLM‐RPA‐MRN constitute two DNA end resection machineries for human DNA break repair. Genes and Development, 25(4), 350–362. 10.1101/gad.2003811 21325134 PMC3042158

[humu23776-bib-0036] Niu, H. , Chung, W. ‐h , Zhu, Z. , Kwon, Y. , Zhao, W. , Chi, P. , … Sung, P. (2010). Mechanism of the ATP‐dependent DNA end resection machinery from *S. cerevisiae* . Nature, 467(7311), 108–111. 10.1038/nature09318 20811460 PMC2955862

[humu23776-bib-0037] O'Driscoll, M. , Ruiz‐Perez, V. L. , Woods, C. G. , Jeggo, P. A. , & Goodship, J. A. (2003). A splicing mutation affecting expression of ataxia‐telangiectasia and Rad3‐related protein (ATR) results in Seckel syndrome. Nature Genetics, 33(4), 497–501. 10.1038/ng1129 12640452

[humu23776-bib-0038] Ogi, T. , Walker, S. , Stiff, T. , Hobson, E. , Limsirichaikul, S. , Carpenter, G. , … Jeggo, P. A. (2012). Identification of the first ATRIP‐deficient patient and novel mutations in ATR define a clinical spectrum for ATR‐ATRIP seckel syndrome. PLOS Genetics, 8(11), e1002945 10.1371/journal.pgen.1002945 23144622 PMC3493446

[humu23776-bib-0039] Phowthongkum, P. , & Sun, A. (2017). Novel truncating variant in DNA2‐related congenital onset myopathy and ptosis suggests genotype–phenotype correlation. Neuromuscular Disorders, 27, 616–618. 10.1016/j.nmd.2017.03.013 28554558

[humu23776-bib-0040] Pires, D. E. V. , Blundell, T. L. , & Ascher, D. B. (2016). MCSM‐lig: Quantifying the effects of mutations on protein‐small molecule affinity in genetic disease and emergence of drug resistance. Scientific Reports, 6, 1–8. 10.1038/srep29575 27384129 PMC4935856

[humu23776-bib-0041] Qvist, P. , Huertas, P. , Jimeno, S. , Nyegaard, M. , Hassan, M. J. , Jackson, S. P. , & Borglum, A. D. (2011). CtIP Mutations Cause Seckel and Jawad Syndromes. PLOS Genetics, 7(10), e1002310 10.1371/journal.pgen.1002310 21998596 PMC3188555

[humu23776-bib-0042] Reynolds, J. J. , Bicknell, L. S. , Carroll, P. , Higgs, M. R. , Shaheen, R. , Murray, J. E. , … Stewart, G. S. (2017). Mutations in DONSON disrupt replication fork stability and cause microcephalic dwarfism. Nature Genetics, 49(4), 537–549. 10.1038/ng.3790 28191891 PMC5450907

[humu23776-bib-0043] Ronchi, D. , Di Fonzo, A. , Lin, W. , Bordoni, A. , Liu, C. , Fassone, E. , … Comi, G. P. (2013). Mutations in DNA2 link progressive myopathy to mitochondrial DNA instability. American Journal of Human Genetics, 92(2), 293–300. 10.1016/j.ajhg.2012.12.014 23352259 PMC3567272

[humu23776-bib-0044] Schymkowitz, J. , Borg, J. , Stricher, F. , Nys, R. , Rousseau, F. , & Serrano, L. (2005). The FoldX web server: An online force field. Nucleic Acids Research, 33, W382–W388. (Web Server issue). 10.1093/nar/gki387 15980494 PMC1160148

[humu23776-bib-0045] Seckel, H. P. G. (1960). Bird‐headed dwarfs: studies in developmental anthropology including human proportions. Basel & New York: S. Karger.

[humu23776-bib-0046] Shaheen, R. , Faqeih, E. , Ansari, S. , Abdel‐Salam, G. , Al‐Hassnan, Z. N. , Al‐Shidi, T. , … Alkuraya, F. S. (2014). Genomic analysis of primordial dwarfism reveals novel disease genes. Genome Research, 24(2), 291–299. 24389050 10.1101/gr.160572.113 PMC3912419

[humu23776-bib-0047] Shaheen, R. , Maddirevula, S. , Ewida, N. , Alsahli, S. , Abdel‐Salam, G. M. H. , Zaki, M. S. , … Alkuraya, F. S. (2018). Genomic and phenotypic delineation of congenital microcephaly. Genetics in Medicine, 21, 545–552. 10.1038/s41436-018-0140-3 30214071 PMC6986385

[humu23776-bib-0048] Singh, G. , & Cooper, T. A. (2006). Minigene reporter for identification and analysis of cis elements and trans factors affecting pre‐mRNA splicing. Biotechniques, 41(2), 177–181. 10.2144/000112208 16925019

[humu23776-bib-0049] Sturzenegger, A. , Burdova, K. , Kanagaraj, R. , Levikova, M. , Pinto, C. , Cejka, P. , & Janscak, P. (2014). DNA2 cooperates with the WRN and BLM RecQ helicases to mediate long‐range DNA end resection in human cells. Journal of Biological Chemistry, 289(39), 27314–27326. 10.1074/jbc.M114.578823 25122754 PMC4175362

[humu23776-bib-0050] Thangavel, S. , Berti, M. , Levikova, M. , Pinto, C. , Gomathinayagam, S. , Vujanovic, M. , … Vindigni, A. (2015). DNA2 drives processing and restart of reversed replication forks in human cells. Journal of Cell Biology, 208(5), 545–562. 10.1083/jcb.201406100 25733713 PMC4347643

[humu23776-bib-0051] Verloes, A. , Drunat, S. , Gressens, P. , & Passemard, S. (1993). Primary autosomal recessive microcephalies and seckel syndrome spectrum disorders In Adam M. P., Ardinger H. H., Pagon R. A., Wallace S. E., Bean L. J. H., Stephens K., & Amemiya A. (Eds.), GeneReviews((R)). Seattle: University of Washington. 20301772

[humu23776-bib-0052] Vetro, A. , Savasta, S. , Russo Raucci, A. , Cerqua, C. , Sartori, G. , Limongelli, I. , … Zuffardi, O. (2017). MCM5: A new actor in the link between DNA replication and Meier‐Gorlin syndrome. European Journal of Human Genetics, 25(5), 646–650. 10.1038/ejhg.2017.5 28198391 PMC5437912

[humu23776-bib-0053] Waterhouse, A. M. , Procter, J. B. , Martin, D. M. , Clamp, M. , & Barton, G. J. (2009). Jalview Version 2—A multiple sequence alignment editor and analysis workbench. Bioinformatics, 25(9), 1189–1191. 10.1093/bioinformatics/btp033 19151095 PMC2672624

[humu23776-bib-0054] Zheng, L. , Zhou, M. , Guo, Z. , Lu, H. , Qian, L. , Dai, H. , … Shen, B. (2008). Human DNA2 is a mitochondrial nuclease/helicase for efficient processing of DNA replication and repair intermediates. Molecular Cell, 32(3), 325–336. 10.1016/j.molcel.2008.09.024 18995831 PMC2636562

[humu23776-bib-0055] Zhou, C. , Pourmal, S. , & Pavletich, N. P. (2015). Dna2 nuclease‐helicase structure, mechanism and regulation by Rpa. eLife, 4, 1–19. 10.7554/eLife.09832 PMC4716839 26491943

