# Supplementary material for: Biallelic variants in DNA2 cause microcephalic primordial dwarfism
Source: Hum Mutat. 2019 Jun 23;40(8):1063–70. doi: 10.1002/humu.23776 (PMC6773220; doi:10.1002/humu.23776)
Supplement: Supplementary file 1 — Supporting information [file HUMU-40-1063-s001.docx]

**SUPPORTING INFORMATION**

**Biallelic variants in *DNA2* cause microcephalic primordial dwarfism**

Žygimantė Tarnauskaitė^1^, Louise S. Bicknell^1,#^, Joseph A. Marsh^1^, Jennie E. Murray^1^, David A. Parry^1^, Clare V. Logan^1^, Michael B. Bober^2^, Deepthi C. de Silva^3^, Angela L. Duker^2^, David Sillence^4,5^, Carol Wise^6,7,8,9^, Andrew P. Jackson^1^*, Olga Murina^1^, Martin A.M. Reijns^1^

∗Correspondence to: Andrew P. Jackson, MRC Human Genetics Unit, MRC Institute of Genetics and Molecular Medicine, Western General Hospital, Crewe Road, Edinburgh EH4 2XU, United Kingdom. E-mail: andrew.jackson@igmm.ed.ac.uk

**Contents:**

- Materials and methods

- Supp. Figure S1. The DNA2-T655A MPD variant is predicted to have reduced ADP binding affinity, just like K654E and K654R, substitutions that abolish DNA2 ATPase activity

- Supp. Table S1. Novel DNA2 variants in four MPD patients and their phenotypic characteristics

- Supp. Table S2. Clinical data for MPD patients with DNA2 variants

- Supp. Table S3. Additional rare sequence variants identified through whole exome sequencing present in Development Disorder Genotype - Phenotype Database (DDG2P) genes

- Supp. Table S5-7 Oligonucleotides

- Supplementary references

**Materials and methods**

**Research subjects**

Patients were recruited to research studies at the MRC Human Genetics Unit in Edinburgh, UK and the Nemours Foundation, Delaware, USA by their local clinician. USA patients were enrolled in the University of Texas Southwestern Medical Center Majewski Osteodysplastic Primordial Dwarfism Type II (MOPD II) DNA/Mutation Registry. The research studies were approved by the Multicentre Research Ethics Committee for Scotland (05/MRE00/74), the Nemours Office of Human Subject Protection (NOHSP) and Institutional Review Board, and the University of Texas Southwestern Medical Center Institutional Review Board respectively. Informed consent was obtained from all participating families. Parents provided written consent for the publication of photographs of the affected individual, P1.

**WES Cohort definition**

Inclusion criteria for recruitment to the study were based on phenotypic criteria of OFC ≤

-4sd, and height ≤ -4sd. Cases were excluded if a cytogenetic abnormality or environmental aetiology had been identified during clinical diagnostic investigations. Prior to WES, where appropriate, targeted Sanger sequencing was performed to exclude syndromic forms of MPD (MOPD I, MOPD II, MGS, etc).

**Exome sequencing and variant validation**

Genomic DNA was extracted from peripheral blood by standard methods or saliva samples using Oragene collection kits according to manufacturer’s instructions. Whole exome capture and sequencing for P1 and P3 was performed at the Wellcome Trust Sanger Institute (WTSI), UK, as previously described (Murray et al., 2014). WES for P4 was performed at Edinburgh Genomics. For the latter, DNA was sheared (Covaris S2), exome capture performed using the Agilent SureSelect v5 enrichment kit according to manufacturer’s instructions, and libraries were sequenced with 125 bp read-pairs using the Illumina HiSeq 2500 V4 platform. All analyses were performed as previously described (Murray et al., 2014).

Variants were confirmed by bidirectional capillary dye-terminator sequencing and annotated using the reference sequence, GenBank: NM_001080449.2 (MIM# 601810). Capillary sequencing was performed in the MRC Human Genetics Unit, Edinburgh, UK. Primer sequences and PCR conditions for targeted *DNA2* sequencing are available on request.

**CADD scores**

Variants were mapped to GRCh38 coordinates (GRCh38-v1.4) and scored with Combined Annotation-Dependent Depletion, CADD (Kircher et al., 2014), using a local instance of the CADD scoring scripts (<https://github.com/kircherlab/CADD-scripts>).

**Splice predictions**

The effects of intronic variants on pre-mRNA splicing were predicted using Alamut® Visual Interactive Biosoftware v2.1.1 (Softgenetics). Alamut Visual uses five distinct splice site prediction algorithms: SpliceSiteFinder-like, MaxEntScan, NNSPLICE, GeneSplicer, and Human Splicing Finder (Desmet et al., 2009; Pertea, Lin, & Salzberg, 2001; Reese, Eeckman, Kulp, & Haussler, 1997; Shapiro & Senapathy, 1987; Yeo & Burge, 2004).

**Minigene splicing assay**

The splicing minigene reporter RHCglo (Singh & Cooper, 2006), a kind gift from T. Cooper, was used for minigene splicing assays. *DNA2* exons and introns of interest were amplified from control and patient genomic DNA using primers with restriction enzyme sites (***Supp. Table S5***) and cloned into RHCglo using standard molecular biology methods. Positive controls for aberrant splicing were generated by introducing a point mutation into donor or acceptor splice sites using QuikChange Site-Directed Mutagenesis (Stratagene) and primers indicated in ***Supp. Table S6***.

HeLa cells, used for transient minigene transfections, were plated in 6-well plates and transfected with 800 ng of minigene plasmids using Lipofectamine™ 2000 (Invitrogen). Cells were harvested 24 h after transfection, followed by total cellular RNA extraction using the RNeasy Mini Kit (Qiagen) and cDNA generation SuperScript™ III Reverse Transcriptase (Invitrogen). PCR amplification with RHCglo or gene-specific primers (***Supp. Table S7***) was then performed, and wild-type and mutant cDNA amplicons were resolved on 2% agarose gels to visualise splicing differences. PCR products were cloned into pGEM-T Easy Vector (Promega), and multiple individual clones Sanger sequenced.

**Structural analysis and *in silico* mutagenesis**

The empirical forcefield FoldX version 4 (Guerois, Nielsen, & Serrano, 2002; Schymkowitz et al., 2005) was used to assess the difference in calculated free energy of folding (i.e. stability change, ΔΔG) between wild-type and mutant DNA2, using all default parameters. The crystal structure of mouse DNA2 bound to ADP (PDB ID: 5EAN; (Zhou, Pourmal, & Pavletich, 2015)) was used. The FoldX ‘RepairPDB’ command was first run on the structure, followed by the ‘BuildModel’ function in order to calculate the change in stability, using all default parameters and 50 replicates. The effect of the human UPF1 subsitutions p.K509R, p.K509E and p.T510A and S. cerevisae SEN1 substitutions p.K1364E, p.K1364R and p.T1364A (equivalent to human DNA2 substitutions p.K654E, p.K654R and p.T655A, respectively) on ADP binding affinity was determined using the crystal structures of UPF1 (PDB ID: 2XZO; (Chakrabarti et al., 2011)) and SEN1 (PDB ID: 5MZN; (Leonaite et al., 2017)) with Mg^2+^ and ADP bound. The effect on protein-ligand affinity was quantified using the mCSM-lig method (Pires, Blundell, & Ascher, 2016), assuming a wild-type affinity (*K*_d_) of 1 nM. PyMol (The PyMOL Molecular Graphics System, Schrödinger LLC; http://www.pymol.org) was used for 3D visualisation, analysis and preparation of structure-based figures.


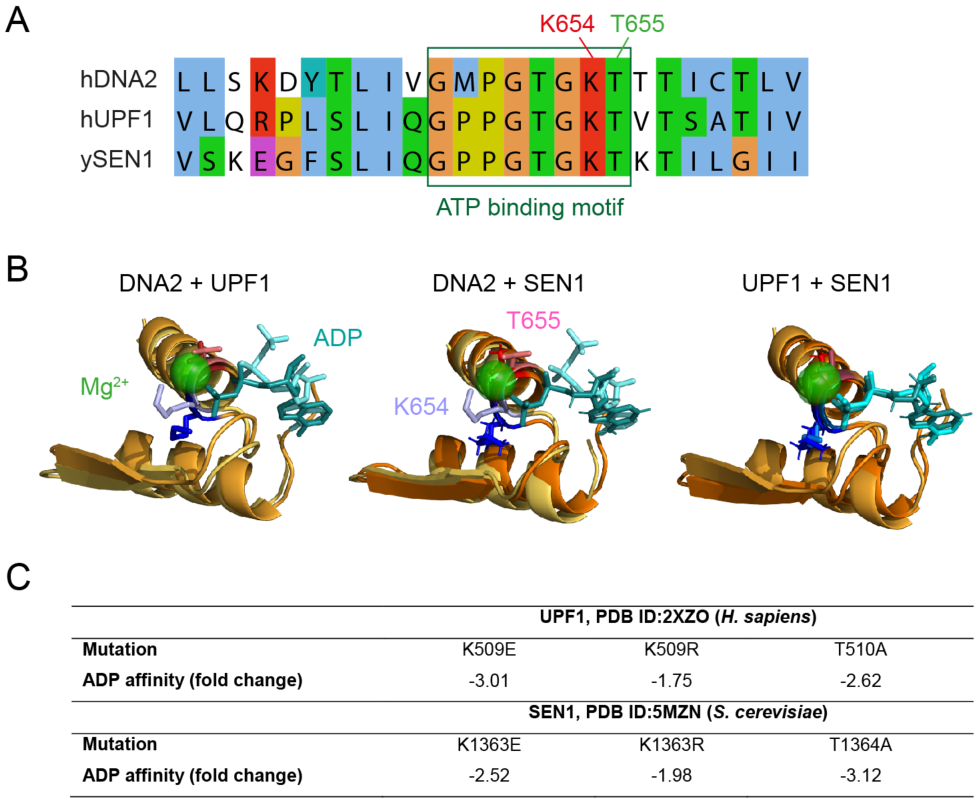


**Supp. Figure S1. The DNA2-T655A MPD variant is predicted to have reduced ADP binding affinity, just like K654E and K654R, substitutions that abolish DNA2 ATPase activity**

**A:** The amino acid sequence of the human DNA2 ATP-binding site is highly conserved in human UPF1 (UniProtKB, Q92900) and yeast SEN1 (UniProtKB, Q00416) helicases. Alignment generated using Clustal Omega with default settings in the Jalview multiple sequence alignment software (Waterhouse, Procter, Martin, Clamp, & Barton, 2009).

**B:** High structural conservation at the DNA2, UPF1 and SEN1 ATP-binding sites, but differential contacts between conserved lysine and threonine residues with ADP due to the presence of a magnesium ion, essential for ATPase activity. Structural overlays of the indicated ATP-binding sites: DNA2 (PDB ID: 5EAN; (Zhou et al., 2015)) in the lightest shade, UPF1 (PDB ID: 2XZO; (Chakrabarti et al., 2011)) in an intermediate shade and SEN1 (PDB ID: 5MZN; (Leonaite et al., 2017)) in the darkest shade. Note that the magnesium ion (green sphere) is only present in the UPF1 and SEN1 structures changing the orientation of ADP and contacts between the lysine (blue) and threonine (red/pink) residues and ADP (cyan). Light blue, pink = DNA2. Dark blue, red = UPF1/SEN1.

**C:** Reduced ADP binding affinity due to lysine to glutamate and threonine to alanine substitutions. The effect of amino acid substitutions on protein-ligand affinity was quantified using the mCSM-lig method (Pires et al., 2016) and UPF1 and SEN1 crystal structures bound to Mg^2+^ and ADP. UPF1-K509/T510 and SEN1-K1363/T1364 are homologous to DNA2-K654/T655.

**Supp. Table S1. Novel *DNA2* variants in four MPD patients and their phenotypic characteristics**

|  | **Nucleotide substitutions / indels**^†^ | | **Amino acid alterations**^‡^ | **Sex** | **Country  of origin** | **Birth** | | | | | **Postnatal** | | | | |
| --- | --- | --- | --- | --- | --- | --- | --- | --- | --- | --- | --- | --- | --- | --- | --- |
|  | **Allele 1** | **Allele 2** |  |  |  | **Gestation (weeks)** | **Weight (kg)** | **SD** | **OFC (cm)** | **SD** | **Age at exam** | **OFC (cm)** | **SD** | **Height (cm)** | **SD** |
| P1 | c.1764-38_1764-37ins(53) | c.1764-38_1764-37ins(53) | p.(Ser588 ArgfsTer4) | F | Italy | 36 | 1.68 | -2.5 | NA^§^ | NA | 5y 8m^¶^ | 43 | -7.5 | 88 | -5.3 |
| P2 | c.1764-38_1764-37ins(53) | c.1764-38_1764-37ins(53) | p.(Ser588 ArgfsTer4) | F | USA | 38 | 1.56 | -3.7 | NA | NA | Adult | 48.8 | -6.5 | 115 | -8.0 |
| P3 | c.74+4A>C | c.1963A>G | p.? / p.Thr655Ala | F | USA | 34 | 0.9 | -4.1 | NA | NA | 15y 9m | 42.1 | -9.6 | 95.5 | -11 |
| P4 | c.74+4A>C | c.74+4A>C | p.? | M | Sri Lanka | 40 | 1 | -5.9 | 29 | -4.9 | 10y 7m | 45.5 | -5.7 | 112 | -4.6 |

^†^ Biallelic variants in *DNA2* (NM_001080449.2; MIM# 601810) found during WES analysis or targeted sequencing of MPD patients (P1–P4). Sequencing of parents of P1, P4, mother of P3; and unaffected 3 siblings of P1 and single sibling of P3, consistent with segregation of an autosomal recessive disorder. Mother of P3 carries the c.74+4A>C variant but not the c.1963A>G change, which was inferred to be on the paternal allele (paternal DNA unavailable).

^‡^ Predicted consequences at protein level; ^§^ NA = Information not available; ^¶^ y = years; m = months

**Supp. Table S2. Clinical data for MPD patients with *DNA2* variants**

| **Patient** | **Gender** | **Age** | **Parental Consanguinity** | **Height (SD)** | **OFC (SD)** | **Cognition**^†^ | **Other information** |
| --- | --- | --- | --- | --- | --- | --- | --- |
| P1 | F | 5y 8m | Yes | -5.3 | -7.5 | Normal | Prominent nose with shallow paranasal groove. Short columella, high frontal hairline. Icthyotic skin over legs. MRI brain, normal. |
| P2 | F | Adult | NA | -8.0 | -6.5 | Normal | Thin, narrow face. Short columella. Large front upper incisors. Marked microtia. Mild micrognathia. Sparse hair frontally, high forehead. |
| P3 | F | 15y 9m | No | -11.1 | -9.6 | Normal | Severe kyphoscoliosis (thoracic), micrognathia, large front upper incisors, recurrent chest infections. MRI brain, no structural abnormalities. Non-specific high T1 signal in basal ganglia. |
| P4 | M | 10y 7m | No | -4.6 | -5.7 | Normal | Protruding large ears, small bud-like mouth, high forehead, broad nasal bridge and tip. Mild micrognathia. Disproportionate short stature (arm span > height). Hyperextensible joints. Scapular winging, lumbar lordosis. Sparse hair. Downslanting palpebral fissures. Mild hepatic enlargement and deranged LFTs. Left orchidoplexy. |

^†^ P1, P3 and P4 in mainstream school, coping well academically. P2, high-school level education attainment. NA, Information not available

**Supp. Table S3. Additional rare sequence variants identified through whole exome sequencing present in Development Disorder Genotype - Phenotype Database (DDG2P) genes.**

| **Patient** | **Position (hg38)** | **Father Genotype** | **Mother Genotype** | **Child Genotype** | **Symbol** | **cDNA consequence** | **Protein Consequence** | **MAF** | **DDG2P disease** |
| --- | --- | --- | --- | --- | --- | --- | --- | --- | --- |
| P3 | chr1:152311503 | NA | NA | T/A  (213,224) | *FLG* | c.3383A>T | p.Glu1128Val | 0.0002924 | Ichthyosis vulgaris |
| P3 | chr1:152314489 | NA | NA | T/C  (145,148) | *FLG* | c.397A>G | p.Asn133Asp | 0.001 |  |
| P4 | chrX:49075539 | G/G  (34,0) | G/A  (23,14) | A  (0,16) | *WDR45* | c.728+6C>T |  |  | Neurodegeneration with brain iron accumulation |

Variants passing GATK hard-filters (FS > 200.0; MQ < 40.0; MQRankSum < -12.5; QD < 2.0; ReadPosRankSum < -20.0) from whole exome sequencing of patients 1, 3 and 4 were filtered for rare (<0.5% allele frequency in public datasets) non-synonymous, loss of function and splice region variants in canonical Ensembl transcripts, not within repeat masked or low complexity regions of the genome, not present in >5% of in-house exome sequenced samples and not present in homozygous or hemizygous state in individuals from gnomAD . Filtered variants were analysed for inheritance consistent with recessive inheritance in each patient requiring a minimum Phred scale genotype quality score of 20, minimum read depth of 10 and alternate allele fraction of 0.25. Variants were also analysed for *de novo* occurrence in P4, for whom parents were also sequenced. All variants that fulfilled these criteria are summarised in Supp. Table S4. Those variants in genes from the DDG2P database (25/1/2019 release) are summarised here. For P1, no variants in DDG2P genes matched these criteria. Genotypes are given with the read depths for reference and alternative alleles given in brackets. cDNA and predicted protein consequences are given relative to Ensembl’s canonical transcript for each gene. NA, Information not available; MAF, Maximum Allele Frequency (gnomAD and 1000 genomes)

**Supp. Table S5. Oligonucleotides used for generating minigene plasmids.**

| **Oligo**  **nucleotide** | **Sequence (5'-3')** | **Description** |
| --- | --- | --- |
| dna2-SalI-int10-F | TTTAATAGCTAGTCGACGGCCCATTCATTCATTCTTTTAGC | Forward primer to clone DNA2 exon 10, intron 11, exon 12, intron 12 and exon 13 into RHCglo |
| dna2-XbaI-int13-R | AGGATGTAATATTTCTAGATCTGAACTGAGGAGAAGCTGATAC | Reverse primer to clone DNA2 exon 10, intron 11, exon 12, intron 12 and exon 13 into RHCglo |
| dna2-SalI-5utr-F | ACGCGTCGACCATTTGGGACATCTGCGGG | Forward primer to clone DNA2 exon 1, intron 1 and exon 2 into RHCglo |
| dna2-XbaI-int2-R | CTAGTCTAGACCCTTAAGATTCAAATCGGTG | Reverse primer to clone DNA2 exon 1, intron 1 and exon 2 into RHCglo |

**Supp. Table S6. Oligonucleotides used for site-directed mutagenesis of minigene plasmids.**

| **Oligonucleotide** | **Sequence (5'-3')** | **Description** |
| --- | --- | --- |
| dna2-c.1764-1A>G F | AGTCAATAATTAAATCTCGAAGTTTTTTGTTGAAAAGTGAAAAAGCACTTTTAGTAATA | Forward primer to introduce c.1764-1A>G mutation in the splice acceptor site of *DNA2* intron 11 |
| dna2-c.1764-1A>G R | TATTACTAAAAGTGCTTTTTCACTTTTCAACAAAAAACTTCGAGATTTAATTATTGACT | Reverse primer to introduce c.1764-1A>G mutation in the splice acceptor site of *DNA2* intron 11 |
| dna2-c.74+1G>A F | CTGCCGGCGGAGCTATGAGCGGAGAGGG | Forward primer to introduce c.74+1G>A mutation in the donor splice site of *DNA2* intron 1 |
| dna2-c.74+1G>A R | CCCTCTCCGCTCATAGCTCCGCCGGCAG | Reverse primer to introduce c.74+1G>A mutation in the donor splice site of *DNA2* intron 1 |

**Supp. Table S7. Oligonucleotides used for RT-PCR analysis of minigene plasmids**

| **Oligonucleotide** | **Sequence (5'-3')** | **Description** |
| --- | --- | --- |
| RSV5U | CATTCACCACATTGGTGTGC | Forward primer in the artificial exon of RHCglo to detect splicing alterations of minigenes |
| RTRHC | GGGCTTTGCAGCAACAGTAAC | Reverse primer in the artificial exon of RHCglo to detect splicing alterations of minigenes |
| dna2-ex11-F | TTGTCGGTCCTTCCAGAATC | Forward primer in DNA2 exon 11 used for RT-PCR |
| dna2-ex13-R | CATCGCTTGCCTCTGAGGC | Reverse primer in DNA2 exon 13 used for RT-PCR |

**Supplementary References**

Chakrabarti, S., Jayachandran, U., Bonneau, F., Fiorini, F., Basquin, C., Domcke, S., . . . Conti, E. (2011). Molecular mechanisms for the RNA-dependent ATPase activity of Upf1 and its regulation by Upf2. *Mol Cell, 41*(6), 693-703. doi:10.1016/j.molcel.2011.02.010

Desmet, F. O., Hamroun, D., Lalande, M., Collod-Beroud, G., Claustres, M., & Beroud, C. (2009). Human Splicing Finder: an online bioinformatics tool to predict splicing signals. *Nucleic Acids Res, 37*(9), e67. doi:10.1093/nar/gkp215

Guerois, R., Nielsen, J. E., & Serrano, L. (2002). Predicting changes in the stability of proteins and protein complexes: A study of more than 1000 mutations. *Journal of Molecular Biology, 320*(2), 369-387. doi:10.1016/S0022-2836(02)00442-4

Kircher, M., Witten, D. M., Jain, P., O'Roak, B. J., Cooper, G. M., & Shendure, J. (2014). A general framework for estimating the relative pathogenicity of human genetic variants. *Nature Genetics, 46*(3), 310-315. doi:10.1038/ng.2892.A

Leonaite, B., Han, Z., Basquin, J., Bonneau, F., Libri, D., Porrua, O., & Conti, E. (2017). Sen1 has unique structural features grafted on the architecture of the Upf1-like helicase family. *EMBO J, 36*(11), 1590-1604. doi:10.15252/embj.201696174

Murray, J. E., Bicknell, L. S., Yigit, G., Duker, A. L., van Kogelenberg, M., Haghayegh, S., . . . Jackson, A. P. (2014). Extreme Growth Failure is a Common Presentation of Ligase IV Deficiency. *Human Mutation, 35*(1), 76-85. doi:10.1002/humu.22461

Pertea, M., Lin, X., & Salzberg, S. L. (2001). GeneSplicer: a new computational method for splice site prediction. *Nucleic Acids Res, 29*(5), 1185-1190.

Pires, D. E. V., Blundell, T. L., & Ascher, D. B. (2016). MCSM-lig: Quantifying the effects of mutations on protein-small molecule affinity in genetic disease and emergence of drug resistance. *Scientific Reports, 6*(March), 1-8. doi:10.1038/srep29575

Reese, M. G., Eeckman, F. H., Kulp, D., & Haussler, D. (1997). Improved splice site detection in Genie. *J Comput Biol, 4*(3), 311-323. doi:10.1089/cmb.1997.4.311

Schymkowitz, J., Borg, J., Stricher, F., Nys, R., Rousseau, F., & Serrano, L. (2005). The FoldX web server: an online force field. *Nucleic Acids Res, 33*(Web Server issue), W382-388. doi:10.1093/nar/gki387

Shapiro, M. B., & Senapathy, P. (1987). RNA splice junctions of different classes of eukaryotes: sequence statistics and functional implications in gene expression. *Nucleic Acids Res, 15*(17), 7155-7174.

Singh, G., & Cooper, T. A. (2006). Minigene reporter for identification and analysis of cis elements and trans factors affecting pre-mRNA splicing. *BioTechniques, 41*(2), 177-181. doi:10.2144/000112208

Waterhouse, A. M., Procter, J. B., Martin, D. M., Clamp, M., & Barton, G. J. (2009). Jalview Version 2--a multiple sequence alignment editor and analysis workbench. *Bioinformatics, 25*(9), 1189-1191. doi:10.1093/bioinformatics/btp033

Yeo, G., & Burge, C. B. (2004). Maximum entropy modeling of short sequence motifs with applications to RNA splicing signals. *J Comput Biol, 11*(2-3), 377-394. doi:10.1089/1066527041410418

Zhou, C., Pourmal, S., & Pavletich, N. P. (2015). Dna2 nuclease-helicase structure, mechanism and regulation by Rpa. *eLife, 4*, 1-19. doi:10.7554/eLife.09832
